# The complete genome sequence of the cold adapted crude-oil degrader: *Pedobacter steynii* DX4

**DOI:** 10.1186/s40793-017-0249-z

**Published:** 2017-07-27

**Authors:** Sijing Chang, Gaosen Zhang, Ximing Chen, Haozhi Long, Yilin Wang, Tuo Chen, Guangxiu Liu

**Affiliations:** 1 University of Chinese Academy of Sciences, No.19A Yuquan Road, Beijing, 100049 China; 2 State Key Laboratory of Cryospheric Sciences, Northwest Institute of Eco-Environment and Resources, Chinese Academy of Sciences, Lanzhou, 730000 China; 3 Key Laboratory of Extreme Environmental Microbial Resources and Engineering, Gansu Province, 730000 China; 4 Key Laboratory of Desert and Desertification, Northwest Institute of Eco-Environment and Resources, Chinese Academy of Sciences, Lanzhou, 730000 China; 5 College of Bioscience and Bioengineering, Jiangxi Agricultural University, Nanchang, Jiangxi 330045 China

**Keywords:** *Pedobacter*, Crude oil, Degradation, Genome

## Abstract

*Pedobacter steynii* DX4 was isolated from the soil of Tibetan Plateau and it can use crude oil as sole carbon and energy source at 15 °C. The genome of *Pedobacter steynii* DX4 has been sequenced and served as basis for analysis its metabolic mechanism. It is the first genome of crude oil degrading strain in *Pedobacter* genus. The 6.58 Mb genome has an average G + C content of 41.31% and encodes 5464 genes. In addition, annotation revealed that *Pedobacter steynii* DX4 has cold shock proteins, abundant response regulators for cell motility, and enzymes involved in energy conversion and fatty acid metabolism. The genomic characteristics could provide information for further study of oil-degrading microbes for recovery of crude oil polluted environment.

## Introduction

The crude oil spills occur frequently and they bring serious pollution to the terrestrial and marine environments [1, 2]. In the bioremediation of crude oil contamination, bacteria work as primary degraders [3–5]. Numerous strains be capable of degrading hydrocarbons have been singled out and identified from marine and terrestrial environments [6–8]. It was also reported that in oil polluted areas, *P*e*dobacter* is one of the major members of alkane degrading bacterial communities [9–11]. For the first time in *Pedobacter* genus, a cultured *Pedobacter cryoconitis* strain was described to have the ability to degrade crude oil [12]. The *Pedobacter steynii* strain DX4 was isolated from frozen soil of Tibetan Plateau permafrost region. This organism was selected for genome sequencing for it exhibited the capability to utilize and degrade crude oil at a cold temperature (15 °C). In this paper, our aim was to identify genomic signatures for petroleum degradation in this strain, and investigate its application in bioremediation in cold environments.

## Organism information

### Classification and features

**Fig. 1:**
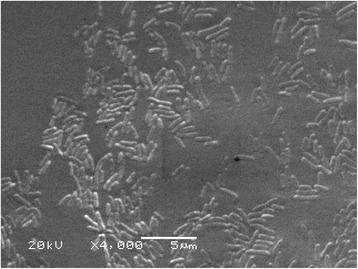
Scanning electron micrograph of *P. steynii* DX4

**Fig. 2:**
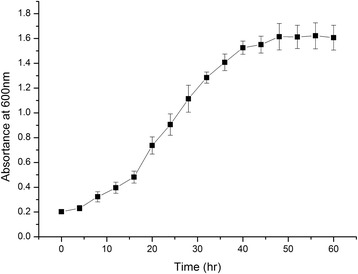
Growth curve of *P. steynii* DX4 in 216 L liquid medium at 20 °C. The absortance at 600 nm was measured every 4 h

**Fig. 3:**
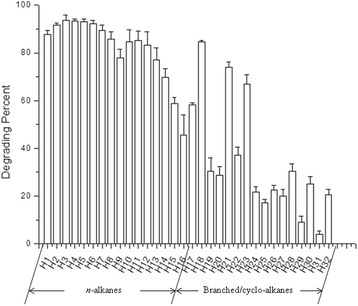
Degrading rates of crude oil by *P. steynii* DX4. H1 - H16: serial *n*-alkanes, from Undecane to Hexacosane. H17- H32: branched alkanes and cycloalkanes, in accordance with the order: Undecane,2,6-dimethyl; Dodecane,2-methyl; Dodecane,2,6,11-trimethyl; Pentadecane, 7-methyl; Octane, 2,3,7-trimethyl; Dodecane, 3-methyl; Dodecane, 2,6,10-trimethyl; 1H-Indene, octahydro-2,2,4,4,7,7-hexamethyl-, *trans*; Undecane, 5-cyclohexyl; H26:Undecane, 4,8-dimethyl; Decahydro-4,4,8,9,10-pentamethylnaphthalene; Pentadecane, 2-methyl; Pentadecane, 2,6,10-trimethyl; Pentadecane, 8-hexyl; Hexadecane; 2,6,10,14-tetramethyl; Ethyl *iso*-allocholate

The soil sample was collected from the Dangxiong County (30.5633°N, 91.4221°E, 4488 m ASL) in the Tibetan Plateau, in 2013. The soil sample was preserved at −20 °C immediately after collection and sent to the State Key Laboratory of Cryospheric Sciences, CAS. The soil type belongs to alpine meadow soil. Crude-oil degrading strains were enriched in liquid MM medium added 2% crude oil (*v*/v) and incubated for 2 weeks at 20 °C [13]. The suspension of culture collection was surface spread onto the 216 L agar plates and cultivated for 5 days at 20 °C [14]. DX4 colonies on 216 L agar plates are light yellow, slightly domed mucoid and circular with smooth margins. DX4 cells are Gram negative rods, motile, non-spore-forming. The scanning electron micrograph is shown in Fig. 1. Additional characteristics of *P. steynii* DX4 are shown in Table 1. Growth experiment was carried out in 216 L liquid medium at 20 °C and the OD_600_ of strain DX4 is shown in Fig. 2. In addition, Fig. 3 shows the crude oil degradation rates of the strain DX4. The degradation was carried out in liquid MM medium added 2% crude oil (*v*/v) at 15 °C for 2 weeks and crude oil was quantified by using gas chromatography and mass spectrometric detector [15].

**Table 1:** Classification and general features of *Pedobacter steynii* DX4

MIGS ID	Property	Term	Evidence code
	Classification	Domain Bacteria	TAS [41]
		Phylum *Bacteroidetes*	TAS [42, 43]
		Class *Sphingobacteriia*	TAS [44–46]
		Order *Sphingobacteriales*	TAS [44]
		Family *Sphingobacteriaceae*	TAS [47, 48]
		Genus *Pedobacter*	TAS [49]
		Species *Pedobacter steynii*	TAS [49]
		Strain DX4	
	Gram stain	Negative	TAS [49]
	Cell shape	Rod	IDA
	Motility	Motile	TAS [49]
	Sporulation	Non-sporulating	TAS [49]
	Temperature range	4-25 °C	IDA
	Optimum temperature	20 °C	TAS [50]
	pH range; Optimum	5-10; 7.5;	IDA
	Carbon source	Yeast extract, pyruvate, crude oil	IDA
MIGS-6	Habitat	Frozen soil	IDA
MIGS-6.3	Salinity	0.5-4.5% NaCl (*w*/*v*)	TAS [51]
MIGS-22	Oxygen requirement	Aerobic	NAS
MIGS-15	Biotic relationship	Free-living	IDA
MIGS-14	Pathogenicity	Non-pathogen	NAS
MIGS-4	Geographic location	China: Tibetan Plateau, Dangxiong County	IDA
MIGS-5	Sample collection	2013	IDA
MIGS-4.1	Latitude	30.5633°N	NAS
MIGS-4.2	Longitude	91.4221°E	NAS
MIGS-4.4	Altitude	4488 m	NAS

^a^Evidence codes – IDA: Inferred from Direct Assay; TAS: Traceable Author Statement (i.e., a direct report exists in the literature); NAS: Non-traceable. Author Statement (i.e., not directly observed for the living, isolated sample, but based on a generally accepted property for the species, or anecdotal evidence). These evidence codes are from the Gene Ontology project

The molecular identification was performed with the 27F-1492R primer to amplify the 16S rRNA sequence. The 16S rRNA from DX4 was 99.64% similar to the *Pedobacter steynii* WB2.3-45^T^ (AM491372) thus DX4 was identified as a strain of *P. steynii*.

**Fig. 4:**
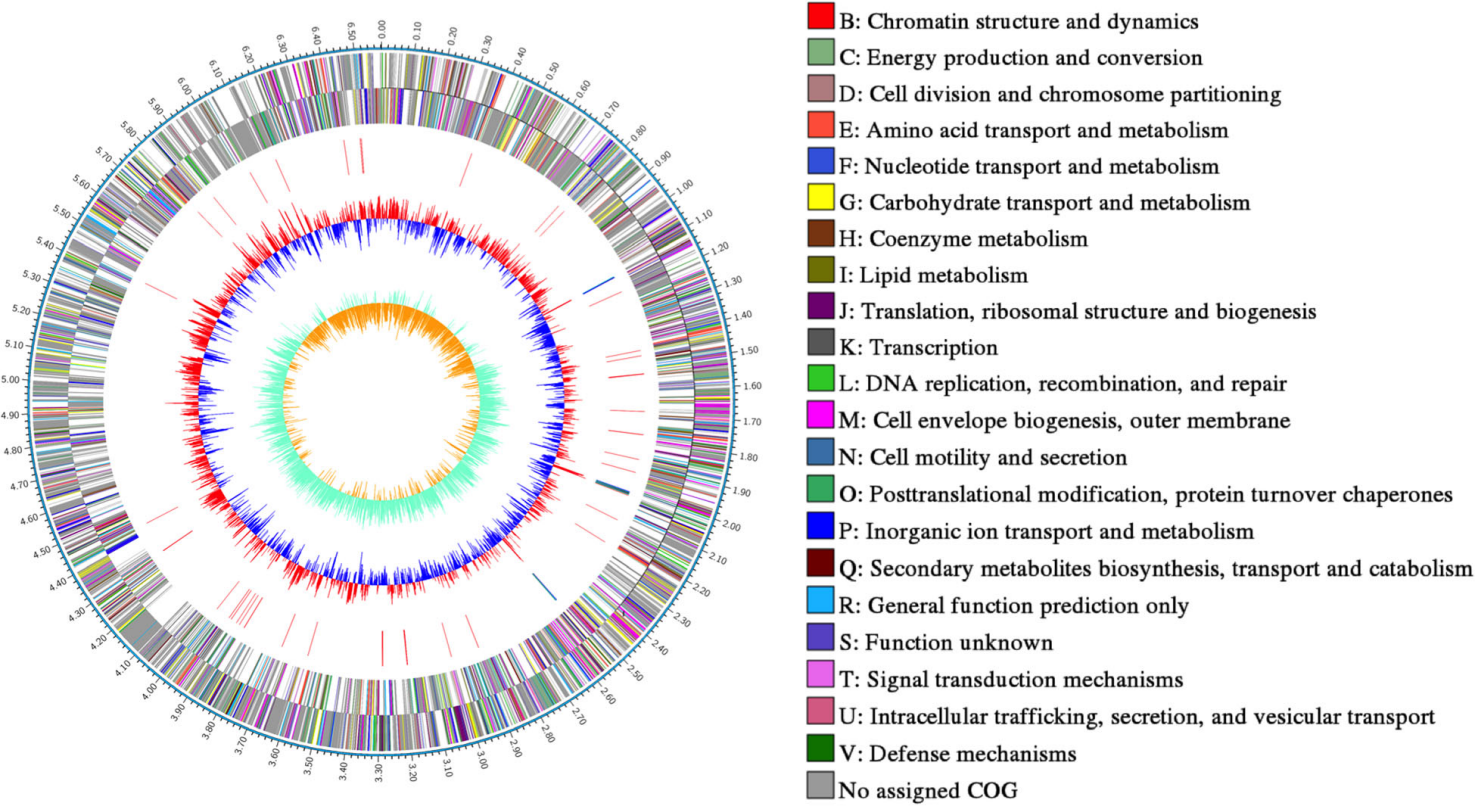
Rooted phylogenetic tree of the 16 S rRNA sequences of *Pedobacter steynii* strain DX4 and relative species. The 16 S rRNA sequences of Pedobacter species were aligned, and the phylogenetic tree was constructed by using Neighbor-joining method based on the maximum composite likehood substitution model

Figure 4 shows the phylogenetic tree constructed from the 16S rRNA sequence together with other related *Pedobacter* species using MEGA 5.0 software suite. The evolutionary history was inferred by using Neighbor-joining method based on the maximum composite likehood substitution model [16, 17].

## Genome sequencing information

### Genome project history

The strain DX4 was selected for sequencing on the basis of its potential biodegradation capability. The initial Illumina sequencing was performed in April 2016 and the genome was closed by PacBio sequencing in August 2016. The genome project is deposited in the online genome database (NCBI-Genome) and the sequence was released for public access on September 9, 2016. A summary of the project information is shown in the Table 2.

**Table 2:** Project information of the whole genome sequence of P. steynii DX4

MIGS ID	Property	Term
MIGS-31	Finishing quality	Finished
MIGS-28	Libraries used	Paired-end (average 500 bp) PacBio (2075 and 2775 kbp)
MIGS-29	Sequencing platforms	Illumina Hiseq 2000 and PacBio
MIGS-31.2	Fold coverage	Illumina paired-end:86× PacBio: 153×
MIGS-30	Assemblers	SOAPdenovo 2.3, GapCloser v1.12 HGAP
MIGS-32	Gene calling method	Glimmer3.02
	Locus Tag	BFS30
	GenBank ID	CP017141
	GenBank Date of Release	September 9, 2016
	GOLD ID	Gp0156107
	BIOPROJECT	PRJNA339039
MIGS-13	Source Material Identifier	DX4
	Project relevance	Biodegrading

### Growth conditions and genomic DNA preparation

*Pedobacter steynii* DX4 was inoculated into 216 L liquid medium and grown on a shaker (200 rpm) at 20 °C, until the cells OD_600nm_ > 1.0. Genomic DNA was extracted from freshly grown cells using the E.Z.N.A.® Bacterial DNA Kit following the standard protocol prescribed by the manufacturer.

### Genome sequencing and assembly

The complete genome sequence of DX4 was sequenced using Illumina HiSeq2000 for the initial sequencing and assembly, followed by PacBio sequencing to fully close the genome sequence [18, 19]. The Illumina platform generated 1,864,026 reads totaling 561,071,826 bp, and the data were assembled into 9 scaffolds by using SOAP denovo V2.3 [20]. The coverage of the paired-end reads was 86×. For gap closure, sequencing was performed using a PacBio SMRT cell, which resulted in 198,008 reads with an average read length of 4973 bp and a coverage of 153×. The alignment of the PacBio reads were assembled with HGAP [21]. Gap closure was managed using the Gap Closer 1.12 and resulting in the final genome of one complete chromosome. This finished genome was deposited in IMG Database with the Project ID: Gp0156107. And this whole-genome project (BioProject ID: PRJNA339039) has also been registered and assembled sequence data submitted at NCBI GenBank under the accession no.CP017141. The Average Nucleotide Identity (ANI) analysis has been carried out by using a online tool [22].

### Genome annotation

Glimmer 3.0 was used to predict open reading frames (ORFs) [23]. The rRNA and tRNA gene predictions and the ORFs annotation were conducted by using BLASTp against NCBI-NR database [24], the COG database [25] and the KEGG database [26]. Genes function annotations were assigned when blastp E-values were ≤0.001 [27]. If there was no significant similarity to protein in other organisms, the gene production was described as hypothetical protein.

## Genome properties

**Fig. 5:**
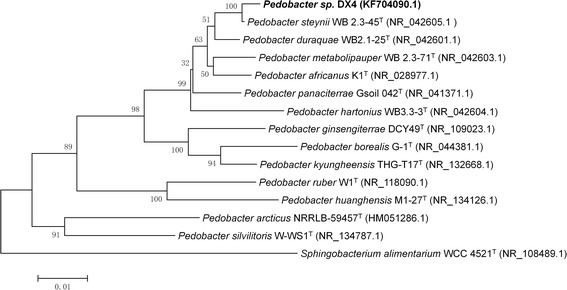
The genome map of *Pedobacter steynii* strain DX4.Circle 1: Base pair numbers; Circle 2 and Circle 3:Forward and reverse coding domain sequences, the color coding of the CDS represent different Clusters of Orthologous Groups categories; Circle 4: rRNA and tRNA; Circle 5: % GC plot; Circle 6:GC skew [(GC)/(G + C)]

The genome statistics is shown in Table 3. The genome of *Pedobacter steynii* DX4 is 6,581,659 base pairs in size, and has a GC content of 41.31%. Out of the total 5464 genes, 23 genes are pseudogenes and 63 are tRNAs, 13 are rRNA genes, 3 are ncRNA genes, 5362 are coding sequences CDSs. Of the total CDSs, 307 are functioning unknown (5.7%), 414 are general function prediction only (7.7%) and the remaining had a defined function. The COG-distribution of genes is shown in Table 4. The genome map (Fig. 5) was visualized by CG view server. The ANI analysis showed *Pedobacter steynii* DX4 had 83.33% nucleotide identity with *Pedobacter steynii* DSM 19110. Comparative analysis between *Pedobacter* strains isolated from polar region was also performed. The *P. steynii* DX4 presented 79.03% nucleotide identity with *P. cryoconitis* PAMC 27485 **(**isolated from Antarctica), 78.42% wit*h* *P. antarcticus* *4BY and* 76.39% with *P. arcticus* A12, revealing the great genetic distance between these strains.

**Table 3:** Genome statistics

Attribute	Value	% of Total
Genome size (bp)	6,581,659	100
DNA coding (bp)	6,033,402	91.67
DNA G + C (bp)	2,718,883	41.31
DNA scaffolds	1	
Total genes	5464	100
Protein coding genes	5362	98.13
RNA genes	79	1.44
Pseudo genes	23	0.42
Genes in internal clusters	NA	
Genes with function prediction	414	7.58
Genes assigned to COGs	3720	68.01
Genes with Pfam domains	4264	78.04
Genes with signal peptides	804	14.71
Genes with transmembrane helices	178	3.26
CRISPR repeats	1

**Table 4:** Number of genes of *Pedobacter steynii* DX4 with the general COG functional categories

Code	Value	% of total^a^	Description
J	155	2.9	Translation, ribosomal structure and biogenesis
A	0	0	RNA processing and modification
K	417	7.8	Transcription
L	146	2.7	Replication, recombination and repair
B	1	0	Chromatin structure and dynamics
D	20	0.4	Cell cycle control, Cell division, chromosome partitioning
V	94	1.8	Defense mechanisms
T	261	4.9	Signal transduction mechanisms
M	306	5.7	Cell wall/membrane biogenesis
N	17	0.3	Cell motility
U	29	0.5	Intracellular trafficking and secretion
O	158	2.9	Posttranslational modification, protein turnover, chaperones
C	150	2.8	Energy production and conversion
G	255	4.8	Carbohydrate transport and metabolism
E	257	4.8	Amino acid transport and metabolism
F	74	1.4	Nucleotide transport and metabolism
H	125	2.3	Coenzyme transport and metabolism
I	154	2.9	Lipid transport and metabolism
P	290	5.4	Inorganic ion transport and metabolism
Q	90	1.7	Secondary metabolites biosynthesis, transport and catabolism
R	414	7.7	General function prediction only
S	307	5.7	Function unknown
-	1642	30.6	Not in COGs

^a^The total is based on the total number of protein coding genes in the genome

## Insights from the genome sequence

Genome annotation predicted many traits support the adaptability of DX4 to cold and crude oil-contaminated environment. The Five cold shock proteins were predicted (NCBI Protein database: WP_069377418.1, WP_062548063.1, WP_048905418.1, WP_008241764.1 and AOM75720.1). These proteins are supposed to play important roles in low temperature conditions [28]. The related strians isolated from antarctic regions, *Pedobacter antarcticus* 4BY and *Pedobacter cryoconitis* PAMC 27485, respectively encoded four cold shock proteins. Based on the COG analysis, 261 genes in total were assigned to the signal transduction category. Among them, 22 genes were predicted to encode the response regulators and 6 were found to encode chemotaxis protein CheY [29]. These genes could play regulatory role in environment sensing and cell motility towards the crude oil.

**Fig. 6:**
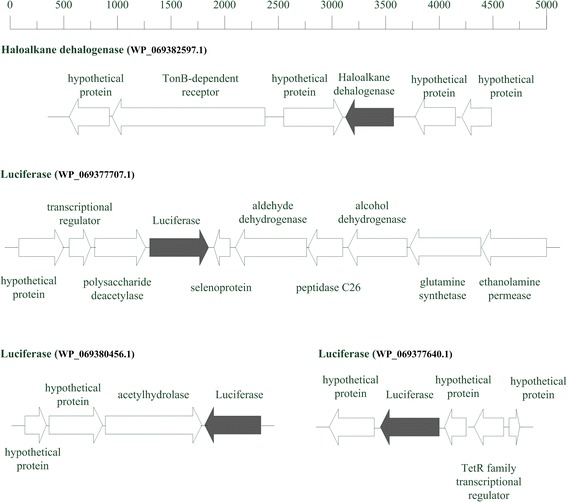
Organization of Genes coding for HLD and luciferase and their adjacent genes in *P. steynii* strain DX4 genome

As for aerobic alkane degradation, alkB gene has been considered as a functional biomarker for alkane-degrading bacterial populations in environmental [30–32]. But in *P. steynii* DX4 genome, no alkB homolog coding genes were found. A gene coding for haloalkane dehalogenase (WP_069382597.1, EC 3.8.1.5) was annotated. Haloalkane dehalogenase (HLD) has considerable environmental significance because it converts haloalkanes to corresponding alcohol and hydrogen halide (KEGG database: RN: R02337,) [33, 34]. In addition to that, three luciferase proteins were identified (WP_069377707.1, WP_069380456.1 and WP_069377640.1). Research showed that the bacteria luciferase can utilize reduced FMN in the oxidation of alkanes with the emission of blue-green light [35, 36]. Figure 6 shows the genes coding for HLD and luciferase protein and adjacent genes upstream and downstream, which may be relevant genes participating in the metabolism of crude oil. In addition, the presence of 19 alcohol dehydrogenase and 23 aldehyde dehydrogenase necessary for alkane degradation as well as 11 fatty acid transport and metabolism genes suggest a complete alkane degradation pathway [37, 38].

The antibiotics and secondary metabolite analysis was done using the anti-SMASH platform [39]. In total, 12 secondary metabolite clusters were identified and 11 of them were related to antibiotics. A resorcinol metabolite cluster was identified and this cluster may play important role in the degradation of resorcinol and other aromatic compounds [40]. Interestingly, the 12 secondary metabolite clusters had no similarity with the known clusters, suggesting that the *P. steynii* strain DX4 may possess novel secondary metabolic pathways.

## Conclusions

*Pedobacter steynii* DX4 was isolated from a cold environment and could utilize crude oil as sole carbon source. The genome of DX4 reported here provides the genetic basis of its crude oil biodegrading mechanism. Genes involved in cold shock, energy conversion and response regulators for cell motility point to the unique abilities of DX4 in oil degradation and cold environment adaptation. Genomic research on DX4 would also provide a blueprint for the application of bioremediation and recovery in cold oil-polluted environments.

## Abbreviation

ANI: Average nucleotide identity
HLD: Haloalkane dehalogenase
